# High‐Density Lignin‐Derived Carbon Nanofiber Supercapacitors with Enhanced Volumetric Energy Density

**DOI:** 10.1002/advs.202100016

**Published:** 2021-05-20

**Authors:** Servann Hérou, Josh J Bailey, Matt Kok, Philipp Schlee, Rhodri Jervis, Dan J. L. Brett, Paul R. Shearing, Maria Crespo Ribadeneyra, Magdalena Titirici

**Affiliations:** ^1^ Department of Chemical Engineering Imperial College Road Kensington London SW7 2AZ UK; ^2^ Electrochemical Innovation Lab Department of Chemical Engineering UCL London WC1E 7JE UK; ^3^ The Faraday Institution Quad One, Becquerel Ave, Harwell Campus Didcot OX11 0RA UK

**Keywords:** densification, electrodes, electrospinning, free‐standing, lignin, microstructure, carbon nanofiber supercapacitors, volumetric capacitance

## Abstract

Supercapacitors are increasingly used in short‐distance electric transportation due to their long lifetime (≈15 years) and fast charging capability (>10 A g^−1^). To improve their market penetration, while minimizing onboard weight and maximizing space‐efficiency, materials costs must be reduced (<10 $ kg^−1^) and the volumetric energy‐density increased (>8 Wh L^−1^). Carbon nanofibers display good gravimetric capacitance, yet their marketability is hindered by their low density (0.05–0.1 g cm^−3^). Here, the authors increase the packing density of low‐cost, free‐standing carbon nanofiber mats (from 0.1 to 0.6 g cm^−3^) through uniaxial compression. X‐ray computed tomography reveals that densification occurs by reducing the inter‐fiber pore size (from 1–5 µm to 0.2–0.5 µm), which are not involved in double‐layer capacitance. The improved packing density is directly proportional to the volumetric performances of the device, which reaches a volumetric capacitance of 130 F cm^−3^ and energy density of 6 Wh L^−1^ at 0.1 A g^−1^ using a loading of 3 mg cm^−2^. The results outperform most commercial and lab‐scale porous carbons synthesized from bioresources (50–100 F cm^−3^, 1–3 Wh L^−1^ using 10 mg cm^−2^) and contribute to the scalable design of sustainable electrodes with minimal ‘dead volume’ for efficient supercapacitors.

## Graphical abstract

This paper presents an innovative method to control the microstructure and the density of bio‐based carbon electrodes for energy‐storage applications. Lignin‐derived nanofibers are first electrospun and then compressed uniaxially at 40 bars. After carbonization, the free‐standing porous carbon materials exhibit densities above 0.6 g cm^−3^ and low void volume, leading to high volumetric capacitance of 130 F g^−3^ in supercapacitors.

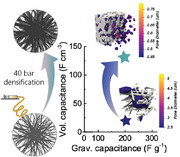

## 1 Introduction

Mitigating climate change is arguably the biggest challenge currently facing humanity; the consequences of which is or will be responsible for loss of biodiversity, population migrations and increasing societal inequalities. The recent transformation of our daily lives due to the COVID 19 pandemic further emphasizes the efforts that need to be applied at the global scale to create a more responsible society, respectful of its environment and inhabitants. As the transportation sector is currently responsible for 15% of the world's greenhouse gas emissions, electrochemical power sources such as supercapacitors(SCs), batteries and fuel cells will play a major role in decarbonizing transport and are required for the transition towards net zero.^[^ ^1^, ^2^, ^3^ ^]^

The main advantages of supercapacitors include their fast charging (seconds), low heat generation and very long lifetime (>100 000 cycles) in comparison with batteries (<2000 cycles).^[^ ^4^, ^5^ ^]^ Thanks to their fast response and low working temperatures, SCs have traditionally been used as high‐power back‐up supply in emergency electronic systems.^[^ ^6^, ^7^, ^8^ ^]^ Their low energy density currently limits their use to start/stop power electronics in electric vehicles (EV), where SCs also improve the efficiency of the propulsion systems, helping to maintain the battery voltage in the desired state‐of‐charge window and to reduce energy drainage.^[^ ^9^ ^]^ SCs are also increasingly used in regenerative braking systems,^[^ ^10^, ^11^ ^]^ which considerably reduce the emission of brake‐related microparticles (e.g., particulate matter smaller than 2.5 µm (PM2.5)) in urban areas whose impact on mortality rates has been proven.^[^ ^12^, ^13^ ^]^ Over the last decade, the energy density of these devices has increased from 5 to 20 Wh kg^−1^ and significant improvements have been reported in the literature of up to 100 Wh kg^−1^ via the design of graphene‐based architectures, surface redox and intercalation pseudo‐capacitive materials with much higher stability than diffusion‐controlled intercalation.^[^ ^14^, ^15^, ^16^ ^]^ These recent improvements in nanostructured electrode materials and highly stable electrolytes make this technology the ideal candidate to power short‐distance electric transportation in urban environments such as buses, trams, ferries, and taxis.^[^ ^5^, ^11^, ^14^ ^]^

Improving compactness and minimizing SC weight is crucial for improving EV efficiency. This is achieved by increasing the loading of active material in the device while maintaining low contact resistance and high capacitance. To accomplish this, two main strategies are employed. The first consists of increasing the thickness of the deposited active layer (µm) on top of the current collector and thus increasing the proportion of the device that is active, rather than inactive, material.^[^ ^17^ ^]^ It involves the design of a microstructure where the electrolyte can easily penetrate to maximize surface utilization.^[^ ^18^ ^]^ In this way, powdered nanocarbons could even be replaced by thick free‐standing electrodes to help further reduce the device weight by 20–30%, making the current collector obsolete.^[^ ^17^, ^19^, ^20^ ^]^ These freestanding materials must exhibit a sufficient electrical conductivity (>1–10 S cm^−1^) both in the electrode planar direction and through the plane, to minimize the Ohmic losses and maintain high cyclability.^[^ ^21^, ^22^, ^23^ ^]^ The second strategy is to increase the packing density of the active material (g cm^−3^), also called the bulk density. This measure of the material's microstructural density must be differentiated from the true density of the carbon framework as measured by helium pycnometry.^[^ ^24^ ^]^ As the packing density considers the volume taken by a specific weight of active material, it takes into account the wide range of porosity scales in the material, from the smallest pore size in carbon structures (≈0.4 nm) to the micrometer range (>50 µm). Recent findings suggest that the presence of porosity greater than 50–100 nm might be ineffective for non‐flow devices such as SCs. This holds particularly true when the electrolyte is able to penetrate the smallest micropores; thus, to enable their usage, it is important to have electrodes with high affinity for the chosen electrolyte.^[^ ^19^ ^]^ In this context, creating a tighter packing of the carbonaceous structure by modifying only the porosity above 50–100 nm enables a reduction of the “dead void volume” without compromising the charge storage capacity.^[^ ^25^ ^]^

Free‐standing graphene films have shown high volumetric performances, combining densities beyond 1 g cm ^−3^ and mass loadings higher than 10 mg cm^−2^.^[^ ^25^, ^26^ ^]^ However, the increase in energy density comes at much higher materials costs^[^ ^27^ ^]^ and raises more environmental concerns than bio‐derived carbons.^[^ ^28^, ^29^ ^]^ Bio‐derived nanostructured carbons are a more sustainable alternative to graphene. They are synthesized from less critical raw materials, such as widely available biomass, from which valuable bio‐polymers (e.g., cellulose, lignins) can be extracted with high degrees of purity.^[^ ^30^ ^]^ Free‐standing lignin derived carbons have shown to combine high energy density and tailored microstructure, allowing electrode flexibility and robustness.^[^ ^31^ ^]^ Despite their low‐cost and abundance, the packing densities of bio‐derived carbons rarely reach values above 0.5 g cm^−3^, which has hindered their commercial viability due to low volumetric energy densities.^[^ ^32^, ^33^, ^34^ ^]^ Despite a few reports focusing on powder compression with binders^[^ ^25^, ^35^ ^]^ and electrode micro‐patterning,^[^ ^32^ ^]^ there is a clear lack of structural engineering strategies to increase the density of bio‐derived carbons while maintaining an efficient pore size for charge storage.^[^ ^36^ ^]^

In this article, a new generation of lignin‐derived carbon nanofiber electrodes is presented, engineered to maximize pore utilization and performance. The low ash content of organosolv lignins (e.g., <1 wt%) yield carbons with very few metal impurities, which decreases the cost of metal removal required for long‐term cyclability.^[^ ^37^ ^]^ Our group has previously demonstrated the production of porous nanofiber electrodes which were binder‐free (self‐standing), electrically conductive and could be easily electrospun using high contents of lignins as renewable precursors.^[^ ^38^, ^39^, ^40^ ^]^ By increasing the points of interfacial contact between the carbon precursor and the porosity generating agent, we were able to optimize the pore size for enhanced double‐layer charge storage.^[^ ^41^, ^42^, ^43^, ^44^ ^]^ However, the intrinsic low packing density of the free‐standing fibrous mats makes them perform poorly on a volumetric basis. We tackle this issue by simply pressing the mats prior to thermal treatment, thus enhancing the volumetric energy density of our lignin‐based carbon nanofiber mats. By using both X‐ray nano‐computed tomography (nano‐CT, which has previously shown to be effective for analyzing the microstructure of electrospun materials^[^ ^45^, ^46^, ^47^ ^]^) and symmetric supercapacitor testing in a full cell, we observe that upon densification, a decrease in micrometer‐size voids improves the volumetric energy density from 0.8 to 6 Wh L^−1^. This renders these electrospun materials ideal candidates for manufacturing high‐density, freestanding, and low‐cost electrodes.

## 2 Results and Discussion

### 2.1 Microstructural Evolution with Applied Pressure

Lignin‐based carbon nanofibers were electrospun from an aqueous alkaline (0.5 m NaOH) lignin:polyethylene oxide (PEO) blending solution containing 90 wt% organosolv hardwood lignin. PEO was used as a plasticizer to enable the electrospinning of lignin without molecular weight fractionation, making the synthesis more scalable and lower cost by avoiding additional steps.^[^ ^48^, ^49^ ^]^

**Figure 1:**
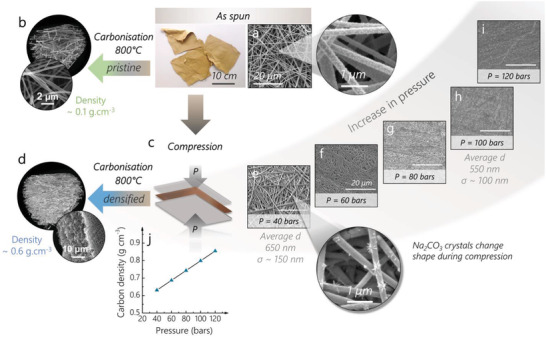
Synthesis scheme for the pristine and densified materials. The as‐spun mat shown in a) the top photograph and SEM micrographs is either carbonized to yield the b) “pristine” sample or c) compressed at various pressures to yield d) the “densified” sample. The SEM of the pressed mat at e–i) various pressures shows the decrease of the surface roughness via nanofiber merging. The pressure applied also promotes growth of the activating agent crystals compared to the as‐spun sample (higher magnification SEM in (e) and (a)). The average diameter and standard deviation, calculated over 30 fibers for the samples compressed at 40 and 100 bars, show that no noticeable variation of the fiber diameter is observed upon compression. j) After carbonization, the bulk density of the densified materials increases linearly with pressure.

The as‐spun polymer nanofiber mats (**Figure** **1**a) were first compressed uniaxially (densified) at pressures between 40 and 120 bars (Figure 1c), then stabilized under air at 200 °C (to improve the charge transfer into the micropores^[^ ^38^ ^]^) and carbonized at 800 °C (Figure 1d). For comparison, analogous mats were thermally treated in the same way but without being pressed (pristine) (Figure 1b).

Electrospun mats exhibit particularly low densities due to the continuous jet of polymer depositing on the collector in a non‐orderly fashion during the electrospinning process (see Figure S1a,b, Supporting Information). The nanofibers stack on top of each other, forming layers and leaving large, micrometer‐sized (1–5 µm) voids within them, which makes up more than 90% of the mat's volume.^[^ ^40^, ^50^ ^]^

Immediately after electrospinning, the mats retain around 10–20 wt% of water (Figure S1d, Supporting Information), which is crucial for plasticizing the polymer blend and facilitating the formation of denser carbon networks upon carbonization.

By compressing the as‐spun mats at room temperature first with 40 bars of pressure, the superimposed nanofiber layers are confined and the contact points between each other increase (Figure 1d,e), bending around one another and filling the voids left during spinning (Figure 1b). Between 60 and 80 bars (Figure 1f,g), the nanofibers start merging, facilitated by the residual amount of water. Beyond 80 bars, the fibrous morphology disappears, and a visibly darker, smoother, denser film forms (Figure 1h,i) as the porosity of the material and surface roughness decrease.^[^ ^51^, ^52^ ^]^

The effects of pressure are also seen in the morphology of the porogenic agent (NaOH), that evolves into Na_2_CO_3_ nanocrystals during the electrospinning process (Figure S1c,e, Supporting Information).^[^ ^41^ ^]^ The shape of the Na_2_CO_3_ crystals changes, from nanodomains that are monodisperse and densely distributed across the surface of the fiber in the no‐pressed sample (Figure 1a), to widely spaced out dendritic domains at 40 bars (Figure 1e). This anisotropic growth is attributed to the pressure‐induced nucleation and growth of the crystals in the direction opposite to the local stress.^[^ ^53^, ^54^ ^]^ This improved interfacial surface area between the porogenic agent and the polymers necessarily causes the development of micro‐porosity, which is beneficial for double‐layer charge storage.

**Figure 2:**
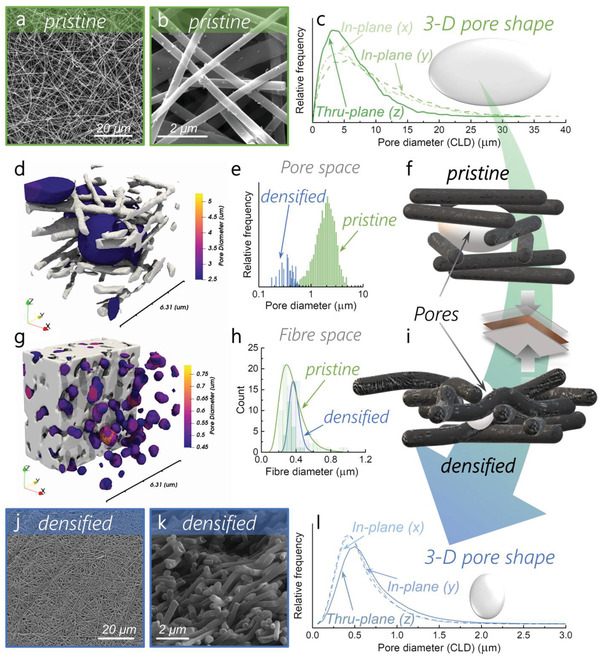
Porosity analysis of the pristine and densified samples. Scanning electron micrographs (SEM) of the a,b) pristine and j,k) densified samples. a,b,j) The top surface of the mats (*x*‐/*y*‐ axis) and k) the side (along the *z*‐axis); 3D Chord Length Distribution (CLD) of the c) pristine and l) densified samples and f,i) corresponding pore shapes, respectively; local thickness results overlaid on fiber volume renderings for the d) pristine and g) densified samples on small scale (≈6 µm); e) pore size distribution determined by local thickness (LT); h) fiber diameter distribution determined by SEM; scheme of the fibrous network in the f) pristine and i) densified samples.

After carbonization, the remaining salt is washed away by soaking the mat in a water bath at 85 °C for 1 h. This dissolves the porogenic agent and opens the micropores of the material, yielding the microporous free‐standing mats. The difference in packing density between the pristine and the densified sample (40 bars) is striking, as observed in the scanning electron micrographs (**Figure** **2**a,b,j,k). The reduction of micrometer‐sized voids observed in pristine is due to the nanofibers bending deformation under the effect of pressure when they encounter one another (Figure 2f,i). However, after compression and pyrolysis (Figure 2j,k) the nanofibers still maintain an overall cylindrical shape. This helps to maintain the presence of small voids and thus is crucial for good penetration of the electrolyte through the electrode thickness. The microstructural changes upon compression affect the surface area of the material in a positive way: the N_2_ sorption isotherms reveal that both the pristine and densified samples are mostly composed of micropores (S_DR_ = 500 m^2^ g^−1^) for the pristine and (S_DR_ = 715 m^2^ g^−1^) for the densified) with pore sizes between 0.5 and 1 nm (Figure S2a,b, Supporting Information).^[^ ^55^ ^]^ These micropores are formed during the etching of the carbon framework by the porogenic agent. The presence of a large microporous volume in the densified sample is in agreement with the larger Na_2_CO_3_ crystals observed in the densified sample due to the effect of pressure (Figure 1a,d). More details on the porosity values are provided in Table S1a, Supporting Information. The development of microporosity is also accompanied by a slightly higher ordering as suggested by the Raman spectra (Figure S2c, Supporting Information) showing a I_d_/I_g_ of 0.9 for pristine and 1.08 for densified. This could be explained by the reorientation of the polymer chains during pressing, facilitated by the residual presence of water in the as‐spun materials.

### 2.2 Microstructure and Void Fraction

To obtain a more representative idea of the electrode densification in three‐dimensions, X‐ray nano‐CT was performed on the densified and pristine materials. Two‐dimensional “ortho‐slices” from these tomograms, along with their segmented counterparts and volume renderings, are provided for more detail in Figure S3, Supporting Information. The decrease of the void fraction after compression can be observed clearly from the volume renderings (Figure 2d,g and Figure S3c,f, Supporting Information), corroborating the densification intuited by SEM (Figure 2a,b,j,k). After careful evaluation of the segmentation to avoid overestimating or underestimating the results, the 3D reconstructions reveal a void fraction of 92% in the pristine sample, which decreases to 21% after compression (densified). The volumetric and areal metrics are provided in Table S1b, Supporting Information. The pore size distribution shifts downwards from having a modal diameter of ≈2 µm in the pristine sample to ≈360 nm for the compacted sample (Figure 2e) in the porosity range that nano‐CT is able to resolve.

The fiber phase (space occupied by fibers) of the samples was analyzed by chord length distribution (CLD)^[^ ^46^ ^]^ and a local thickness (LT) method (see Section 4) and the results are presented both in Figure 2 and Figure S4, Supporting Information. For the pristine sample, the CLD peak lies around 0.40–0.50 µm in all directions (Figure S4a, Supporting Information, corroborating the presence of cylindrical fibers with an average diameter of ≈400–500 nm (Figure 2h). The CLD displays a greater relative frequency in the *z*‐direction, consistent with in‐plane alignment (*x*‐/*y*‐) of the fibers through electrospinning. The LT result (Figure S4b, Supporting Information) also indicates a peak for the fiber diameter ≈400 nm, suggesting that measurement of the fiber morphology is amenable to both approaches.

For the densified sample, although the fibrous nature was still observable by the raw images (orthoslices in Figure S3), the binary image (Figure S3e, Supporting Information) represents a porous mat without definable fibers due to close proximity and overlapping features. As a result, the CLD and LT methods do not give rise to consistent results in the fiber phase for the densified sample. However, a statistical analysis of fiber diameters by SEM along the *z*‐axis (Figure 2h) and (*x*‐/*y*‐) plane (Figure 2a) shows that the diameter of the fibers increases slightly under pressure.

Results from the CLD and LT methods applied to the porous phase of both samples are presented in Figure 2c,l, revealing two clear changes in the pore size distribution upon pressing. First, the peak in the CLD decreases dramatically from 4–5 µm to 0.4–0.5 µm. This represents an order of magnitude decrease in modal chord length and suggests a significant drop in the average pore diameter, as expected upon compaction. Second, a change in anisotropy is observed. In pristine, the *x*‐ and *y*‐distributions are similar, whereas the *z*‐distribution has a higher peak (at slightly lower chord length), as would be expected for an electrospinning process that is random in the *x* and *y* planes but deposits layers of fibers in the *z* plane. However, in densified, the inverse is true, whereby the *z* distribution peaks at a lower relative frequency and slightly longer chord length. These results suggest that upon pressing, not only does the porosity and average pore size significantly decrease, but the pore shape is also affected. The observed trend would be consistent with pores that were initially oblate on average, becoming increasingly spheroidal and potentially prolate after pressing (Figure 2f,i). Although it should be noted that in the very porous case (pristine), an appreciable proportion of chords will likely transect two or more pores, this would be equally likely in all directions should the porous network be truly isotropic.

Interestingly, for the pristine sample, the LT‐derived pore diameter does not match the CLD data as closely as for the densified fibers. The broad peak between 2.0 and 2.5 µm is noticeably smaller than the diameter estimates from CLD (4–5 µm, Figure 2c). This may be a reflection of the methods used. In the case of the CLD approach, multiple “individual pores” are traversed along orthogonal axes, whereas the LT method better segments the connected network into discrete units by virtue of its sphere‐growing procedure, regardless of the orientation of the 3D volume. Nonetheless, the true network is likely to contain pores that deviate from the spherical ideal and thus the average pore size may be more fairly estimated to lie between these limits. In the densified case, a narrower peak at ≈0.36–0.40 µm is observed, in better agreement, and only slightly smaller than the estimated diameter from the CLD approach (0.4–0.5 µm, Figure 2l). Overall, these results show that the average pore size decreases dramatically upon pressing at 40 bars (by somewhere between 12.5 and 3.8 times, on a length basis) and are consistent with pore shape changes from oblate to more spheroidal. Using CLD, the size of these pores was also found to be constant along the whole *z*‐axis (Figure S5a, Supporting Information), indicating that the compression is homogeneous along the whole thickness of the material. The fiber diameter is largely unaffected by the compression and indicates that the fibrous morphology is maintained (Figure S5b, Supporting Information), as observed by SEM. These results show how powerful the CLD and LT methods are to examine both changes in fiber size and shape, and their applicability should be determined by the specific morphology examined. To illustrate the nature of these changes, the results of the LT method on the porous phases of both pristine and densified are overlaid on volume renderings of the samples in each case, as shown in Figure S5c,d, Supporting Information.

### 2.3 Density Implications

The density derived from tomographic analysis was contrasted with the bulk (e.g., weight/volume, accounting for void fraction) and apparent (e.g., skeletal density only) densities for both samples (**Table** **1**). It is worth mentioning that it is the bulk density, *ρ*_t_ (Equation (1), methods) that is commonly used to calculate the volumetric capacitance, while that derived from the N_2_ adsorbed pore volume (*ρ*_DFT_) underestimates the void volume, as it does not take into account the presence of pores larger than 50–100 nm at a maximum relative pressure p/p_0_ ≈0.99 (Equation (2)).^[^ ^24^ ^]^ The density calculated from X‐ray CT (*ρ*_tomo_, Equation (3)) is also largely overestimated as the resolution of the instrument does not allow to resolve the microporosity of the fibers. This technic therefore considers the fibers as solid entities and overestimates the density of the material's skeleton.

**Table 1:** Bulk (*ρ*_t_) and apparent (*ρ*_DFT_) density of pristine and densified materials in comparison to density determined via x‐CT (*ρ*_tomo_)

Electrode	Thickness (*t*) [µm]	Areal density [mg cm^−2^]	*ρ*_*t*_ [g cm^−3^]	*ρ*_DFT_ [g cm^−3^]	*ρ*_tomo_ [g cm^−3^]
Pristine	175 ± 5	1.47	0.1 ± 0.007	1.25	0.16
Densified	50 ± 2	3.10	0.63 ± 0.06	1.20	1.58

The compression step considerably reduces the electrode thickness by ≈125 µm (to 29% of the original), which in turn increases the bulk density abruptly. However, as the apparent density (*ρ*_DFT_) remains constant, the amount of micropores (<2 nm) is similar in pristine and densified and the structure of the charge storing pores is unaffected by the compression. This apparent density is closer to that of dense graphene‐based materials.^[^ ^56^ ^]^ However, these density values are largely over‐estimated, as they omit the presence of pores larger than the ones simulated in the DFT model, including the void structure which makes up the vast majority of free space within the mats. The increase in the apparent density calculated from tomographic analysis (*ρ*_tomo_) also reflects the sharp decrease in void volume upon compression. Due to the increased microporosity in densified (30% higher than in pristine), the apparent density *ρ*_tomo_ in densified reaches a higher compression rate than the bulk density *ρ*_t_. Thus, as the bulk density is improved by a factor of six, the apparent density *ρ*_tomo_ predicts a tenfold increase.

### 2.4 Microstructural Effect on the Electrochemical Performance

The capacitive performance of both materials was tested in a symmetric configuration using a model 6 m KOH electrolyte to show the potential of these materials in a full cell. The absence of binder ensures a good electrical contact between the free‐standing materials and the current collectors, which was assessed by measuring a cell resistance below 1 Ω. The high through‐plane (*z*‐axis) electrical conductivity increased from 0.1 S cm^−1^ for pristine to 0.6 S cm^−1^ for densified which is explained by the increase in electrical contact points between the nanofibers in densified (Figure S6a, Supporting Information). This increase in conductivity in densified is explained by the enhanced surface of contact between the fibers, which facilitates the movement of charges and reduces heat dissipation (Figure S6b, Supporting Information).

**Figure 3:**
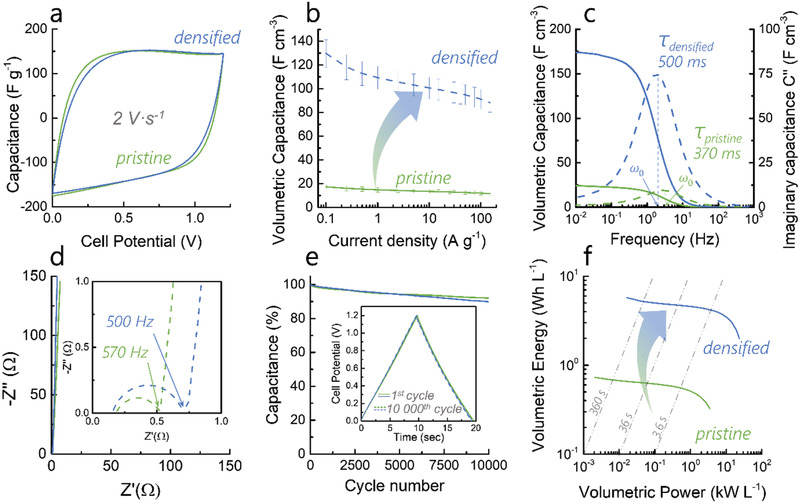
Electrochemical characterization in aqueous 6 m KOH for pristine and densified: a) Cyclic voltammograms measured at 2 V s^−1^; b) volumetric rate capability; c) real and imaginary volumetric capacitance as a function of the frequency obtained by EIS; d) complex plane plots obtained by EIS; (e) cyclability measured at 10 A g^−1^ and f) volumetric Ragone plot showing the performances of pristine and densified.

Pristine and densified show very similar gravimetric capacitances at both low and high current densities, while the gravimetric energy density for both electrodes approaches 10 Wh kg^−1^ (**Figure** **3**a and Figure S8a–c, Supporting Information). This indicates that the compression does not affect the high rate capability of the electrode (75% retention at 100 A g^−1^). After compression, the volumetric capacitance follows the same trend as the bulk density, increasing from 20 to 130 F cm^−3^ at 0.1 A g^−1^ (Figure 3b). Electrochemical impedance spectroscopy (EIS) (Figure 3c,d) shows that the compression has a particular effect on the ion kinetics: densified exhibits a 2.5 higher relaxation time compared with pristine (500 ms vs 199 ms) indicating a slower ionic transference into the micropores. This is also confirmed by the larger charge transfer resistance for densified observed in the Nyquist plot (Figure 3d). This slower ionic mobility is, however, not sufficient to impede the rate capability at the charging rates used commercially (up to 100 A g^−1^) and would probably be observed only at much higher rates. This higher relaxation time in the densified sample could be explained by a slightly less hydrophilic surface due to the enhanced development of microporosity, meaning that less oxidizing agent is available per surrounding carbon atoms. The H_2_O sorption isotherms (Figure S7, Supporting Information) reveal that the densification decreases the wettability of the micropores as the H_2_O adsorbed volume around p/p_0_ ≈ 0.5 decreases from 250 to 200 cm^3^ g^−1^. The polarization of the individual electrodes via galvanostatic charge/discharge (FigureS8d–f, Supporting Information) indicates that the adsorption of cations and anions in pristine and densified follows the same trend. The larger polarization of the working electrode can be explained by the pseudocapacitive contribution of oxygen groups on the counter electrode.^[^ ^38^ ^]^ By analyzing the kinetics of the capacitance, this pseudocapacitance was estimated to contribute to the total capacitance by 14.5% in pristine and 18.3% in densified (Figure S9 and Table S2, Supporting Information).^[^ ^57^ ^]^ This pseudocapacitive contribution, as well as the ion adsorption into the micropores, is also shown to be highly reversible over 10 000 cycles as the symmetric cell maintains over 90% of its capacitance (Figure 3e).

The volumetric Ragone plot shows that the increase in energy density is proportional to the increase in density (Figure 3f). Densified shows a volumetric energy density of 6 Wh L^−1^ at low current densities and still provides 3.4 Wh L^−1^ at 10 kW L^−1^ (Figure 3f). These first results are comparable with non‐free‐standing high‐performing materials produced from biomass and tested in aqueous electrolytes^[^ ^58^ ^]^ or flexible materials such as PEDOT,^[^ ^59^ ^]^ graphene–cellulose composite films^[^ ^60^ ^]^ and activated‐carbon composites.^[^ ^61^ ^]^

**Figure 4:**
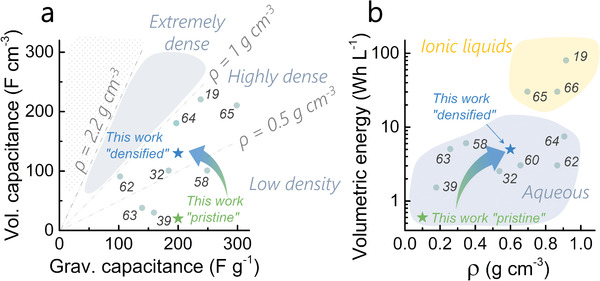
a) Volumetric versus gravimetric capacitance of pristine and densified compared to other works showing the bulk densities classified in three regions (low, high, and extremely high density above 1 g cm^−3^). The density of graphite is show as reference; b) volumetric energy versus bulk density of various materials showing the device energy density obtained to ionic liquid and aqueous electrolytes. The reference numbers are shown for each data point.^[^ ^19^, ^32^, ^39^, ^58^, ^60^, ^62^, ^63^, ^64^, ^65^, ^66^ ^]^

To highlight the main message of this study, the supercapacitive performances of pristine and densified are compared to other dense materials. Electrospinning was demonstrated as a method to nanostructure dense carbon materials from bio‐derived polymers and that compressing the as‐spun mats is the way forward to improve volumetric density. **Figure** **4**a depicts the volumetric and gravimetric capacitance of various materials and their corresponding bulk densities. The tight packing of the nanofiber mats, that is achieved through compression, increases the microporosity of the fibers while reducing the size of non‐storing pores drastically. This produces a sixfold density increase without affecting the gravimetric performance (200 F g^−1^ at 0.1 A g^−1^) (Figure 4a). It also bridges the volumetric performance gap between electrospun materials and other highly dense supercapacitive carbons. As shown on Figure 4b, the compressed electrospun materials can now compete with other dense carbons in terms of device volumetric energy density, for the first time in the literature. These results are compared with graphene‐based electrodes to show that the microporous structure of these free‐standing materials must be now adapted to maintain their high capacitance in high‐voltage electrolytes.

## 3 Conclusion

Supercapacitors are a more sustainable alternative to batteries, depleting fewer natural resources, and are better suited to applications that require low energy densities (10–50 kWh vs >100 kWh for longer‐range vehicles). Free‐standing nanostructured carbon materials with high densities are particularly well‐suited to this technology as they enable compact energy storage whilst decreasing recycling costs. The main advantage of using organosolv lignins for supercapacitor applications is their very low ash contents. This results in carbons with very few metal impurities, thus decreasing the cost of metal removal required for long‐term cyclability. A simple and potentially scalable method is presented to increase the density of electrospun free‐standing mats, usually considered to be of insufficient packing density to provide enough volumetric capacity for supercapacitor applications. Compression at 40 bars decreases the micrometer size void volume of the carbon nanofiber electrodes from 92% to 21%, increasing the packing density by a factor of six. It is shown that the compression is homogeneous on the micrometer scale using X‐ray nano‐computed tomography and the change in pore shapes is characterized. Finally, as a proof‐of‐concept, the potential of these dense electrodes in supercapacitors is demonstrated using 6 m KOH as the electrolyte and the full device shows an energy density of 3 Wh L^−1^ at 10 kW L^−1^. These results aim to serve as a springboard to further improve the density, mass loading and energy density of bio‐derived supercapacitor electrodes by using electrolytes with greater electrochemical stability. It may also benefit the wider community working in electrochemical energy production and storage by providing a simple tool for precisely tuning microstructures in carbon materials.

## 4 Experimental Section

### Reagents

The organosolv lignin was kindly provided by the group of Christine Ro*β*berg at the Fraunhofer Center for Chemical‐Biotechnological Processes (CBP) in Jena. Sodium hydroxide pellets (NaOH, analytical reagent grade) were sourced from Fisher Scientific. Polyethylene oxide (PEO, Mw 600 000 g mol^−1^) was sourced from Sigma‐Aldrich. Both chemicals were used without further purification.

### Synthetic Procedures: Extraction of the Organosolv Lignin

Extracted from beech wood via a water:ethanol organosolv process, this lignin had a sulfur content below 0.5 wt%, a carbohydrate content determined by HSQC‐NMR which was below the detection level, a higher concentration of syringyl than guaicyl units and a molecular weight M_w_/M_n_/PID of 4815/ 3207 g mol^−1^/ 1.50 versus polystyrene (determined by gel permeation chromatography). Details on lignin extraction and characterization can be found in the literature.^[^ ^41^, ^62^ ^]^

### Synthesis of Carbon Nanofibers

The synthesis of the carbon nanofibers was reported in previous reports where more details can be found.^[^ ^41^ ^]^ PEO (0.204 mg) was first dissolved for 2 h in an aqueous NaOH solution (0.5 m, 15 g) by stirring. Then, organosolv lignin (OSL, 1.84 g) was added and the solution was vigorously stirred to yield a polymer concentration of 12 wt% and a ratio PEO:OSL of 1:9. After centrifugation at 10 000 rpm for 5 min, the solution was electrospun in a chamber (Nanobox, Plaslab), where the temperature was maintained at 22 °C and the relative humidity at 25%. The solution was electrospun at a rate of 2 mL h^−1^ using an 18‐gauge needle positively charged at 20 kV. A 25 cm^2^ aluminium collector was electrically grounded and placed 20 cm from the needle. After spinning 4.5 to 5 mL of solution, the mat (approx. 150 µm thickness) was removed from the collector, cut into stripes, sandwiched between two pieces of carbon felt and subjected to heat treatments (MTI 1200x tubular furnace). The stabilization heat treatment was performed under air at 200 °C using a 1 °C min^−1^ heating rate and dwelling time of 2 h. Subsequently, the carbonization was performed at 800 °C for 2 h, using a heating rate of 5 °C min^−1^. After carbonization, the activating salt was washed away by soaking the free‐standing mats in a distilled water bath at 85 °C for 1 h. The textile was then rinsed with ethanol and placed in a vacuum oven at 100 °C to dry overnight.

### Pressing

Densified was fabricated by pressing the as‐spun mats into a denser network. The pressing was achieved by placing two layers of uniform thickness (≈170 µm) of as‐spun mats on top of each other on the bottom plate of a planar hot‐press (Table‐top Platen Press P200E, Dr. Collin GmbH). The mat layers were pressed to 40 ± 5 bars for 10 s, which was enough to stick the two layers together. Due to the pressure applied, the two layers merge into a single one with reduced flexibility compared to the un‐pressed counterpart. This technique permits tunability of the electrode thickness by stacking several layers on top of each other. As the presence of residual humidity in the mats seems to limit the maximum pressure at which the nanofiber mats retain their fibrous morphologies, partially drying the mat before compression could increase the applicable compression force.

### Carbon Material Characterization

The N_2_ sorption isotherms were measured at 77 K using a Quantachrome Autosorb instrument. The relative pressure range was measured between 1 × 10^−5^ and 0.99. This provides information about the pores larger than 0.5 nm. The porosity data (S_BET_, S_DR_, S_BJH_) reported in Table S1, Supporting Information, were calculated by the software Novawin. The BrunauerEmmettTeller surface area (S_BET_) was measured over a relative pressure range of 5 × 10^−3^ to 5 × 10^−2^. The pore size distribution was calculated from the adsorption line using a quenched‐solid model QSDFT assuming slit and cylindrical pores geometries. The mesoporous surface area S_BJH_ was calculated from the adsorption line. The microporous data were calculated from the adsorption line by the Dubinin‐Radushkevic (DR) model on the relative pressure range from 4 × 10^−3^ to 1 × 10^−2^ for pristine and from 5 × 10^−2^ to 3 × 10^−1^ for densified. Scanning electron microscopy (SEM) was used to investigate the microstructures of the as‐spun nanofibers before and after compression. A small but representative amount of sample was fixed onto a steel stub with a sticky carbon tape. All samples were coated with gold (45 s, 15 mA plasma current) prior to imaging to avoid charging artefacts. Images were taken on an FEI Inspect F instrument using an acceleration voltage of 20 kV and a working distance of 10 mm.

### X‐Ray Nano‐CT Acquisition

Tomograms of pristine and the densified samples were acquired on small cylindrical pillars (≈50–100 µm diameter), prepared as discussed elsewhere.^[^ ^67^ ^]^ The tomograms were acquired using a Zeiss Xradia 810 Ultra (Carl Zeiss, Germany) instrument with a fixed Xray source energy of 5.4 keV. To maximize contrast between the lowly attenuating carbon phase and the background air, Zernike phase contrast imaging was employed^[^ ^68^ ^]^ which provided a visual enhancement at the fiber edges. In both cases, binning 1 was used, giving an isotropic voxel dimension of 63 nm in large field‐of‐view (FOV) mode (65 µm FOV). For the as‐spun pristine sample, the exposure time was 64 s and the number of projections 1001. For the denser, slightly larger sample, an increased number of projections (1601) was used to minimize reconstruction artefacts and an exposure time of 30 s gave a total scan time of 15 h.

### X‐Ray Nano‐CT Data Processing

Using Avizo software (2019.2, Thermo Fisher Scientific), subvolumes comprising 548 × 529 × 625 voxels (≈45 500 µm^3^) were extracted from the two 3D tomograms, maximizing the inspected volume while making sure not to capture any void space external to the sample volume. To de‐noise the data, a 3D Gaussian filter was used (kernel size  =  4) before proceeding to segment via a seed‐based watershed approach. This involves conservative “seeding” of both phases based purely on voxel grayscale values and, based on the grayscale gradient, allowing for an “inundation” algorithm to grow these seeds up the sides of the “watershed basins” until they meet and all voxels are allocated. A more detailed description of this segmentation approach can be found here.^[^ ^69^ ^]^

### X‐Ray Nano‐CT Data Analysis

Analysis of the binarized 3D reconstructed data included extracting information on phase fraction, surface area, fiber diameter, pore size distribution and plotting these features in a slice‐by‐slice fashion. Phase fractions and surface areas were calculated in Avizo and all other computational analyses were performed in Python on binarized volumetric images processed as aforementioned.^[^ ^70^ ^]^ A local thickness (LT) approach was used as one approach to calculate the fiber thickness, where each voxel in the fiber phase was assigned the radius of the largest sphere that can be drawn inside that phase and overlapping that voxel.^[^ ^71^ ^]^ Although the fibrous nature was retained after hot‐pressing, due to the high level of compaction and binarization of the data, this approach was only applicable to the pristine sample. Nevertheless, this LT procedure was also applied to the porous phase to provide an estimate of the continuous pore size distribution (cPSD). Furthermore, a chord length distribution (CLD) algorithm was also run in the three principal directions, aligning the *z*‐axis with the direction of the spinning jet, to probe anisotropy and shape in the pristine fibers and pores in both samples. This procedure involves the virtual drawing of chords within the phase of interest (from boundary to boundary) in the *x*‐, *y*‐, and *z*‐directions and by plotting the distribution of their lengths, information about alignment and shape can be accessed. More information about these algorithms can be found here.^[^ ^46^ ^]^

### Electrochemical Measurements

Electrochemical data were obtained using a standard two electrodes symmetric Swagelok cell connected to a VSP‐300 Biologic potentiostat using a Hg/HgO reference electrode (buffer 1 m KCl solution) to record the polarization of both electrodes simultaneously (see Figure S8d–f, Supporting Information). Pristine and densified electrodes were cut from the mat using a hole‐punch with a diameter of 0.7 cm and directly placed on stainless steel current collectors (FigureS10, Supporting Information). Before assembling the cell, the electrodes were wetted by adding a few drops of electrolyte on the electrode and pressing it onto the current collector using a spatula. In this way, the electrolyte can properly infiltrate the macro structure of the electrodes.

### Electrode Density Measurements

The electrode density was calculated using the mass and volume data as follows:

(1) $$\rho_{t}=\frac{m}{t\cdot A}\;\left[g\;cm^{-3}\right]$$

where *m*(g) is the mass, *t*(cm) the thickness and *A*(cm^2^) the surface area of the electrode disc.

(2) $$\rho_{\mathrm{DFT}}=\frac{1}{V_{\mathrm{pore}}+\frac{1}{\rho_{\mathrm{carbon}}}}\;\;\left[g\;cm^{-3}\right]$$

where *V*_pore_(cm^3^) is the pore volume determined by N_2_ adsorption, *ρ*_carbon_ (g cm^−3^) is the density of the carbon structure, estimated to be 2 g cm^−3^.^[^ ^24^ ^]^

(3) $$\rho_{\mathrm{tomo}}=f_{\mathrm{fibers}}\cdot \rho_{\mathrm{carbon}}\;\left[g\;cm^{-3}\right]$$

where *f*_fibers_ is the volume fraction of the fiber phase in the electrode, detailed in Table S1, Supporting Information.

The measurement error on the value of *ρ*_electrode_ can be measured by the logarithmic method

(4) $$\frac{\Delta \rho_{\mathrm{electrode}}}{\rho_{\mathrm{electrode}}}=\frac{\Delta m_{e}}{m_{e}}\;\;+\frac{\Delta t_{e}}{t_{e}}+\frac{\Delta S_{e}}{S_{e}}\;\left[-\right]$$

where Δ*m*_*e*_(g), Δ*t*_*e*_(cm), and Δ*S*_*e*_(cm^2^) are the absolute errors on the mass, the thickness and the surface of the electrode, respectively.

### Electrical Conductivity Measurements

The frequency dependent electrical conductivity of the electrodes was obtained by electrochemical impedance spectroscopy (EIS).

(5) $$\sigma \left(\omega \right)=\sigma^{\prime }\left(\omega \right)+i\sigma^{\prime \prime }\left(\omega \right)\;\;\left[S\;cm^{-1}\right]$$

(6) $$\sigma \left(\omega \right)=\frac{t}{A}\cdot \frac{Z^{\prime }\left(\omega \right)}{{\left|Z\right|}^{2}}+i\frac{t}{A}\cdot \frac{Z^{\prime \prime }\left(\omega \right)}{{\left|Z\right|}^{2}}\;\;\left[S\;cm^{-1}\right]$$

where *ω* (*Hz*) is the frequency, *σ*′(*ω*) is the real component of the electrical conductivity and *σ*′′(*ω*) the imaginary component, *Z*′ (*ω*) and *Z*′′ (*ω*) are the real and imaginary parts of the impedance.

### Cell Assembly

The cells were assembled by pressing the two electrodes separated by a 6 mm disc glass fiber separator (Whattman) between the two current collectors. Prior to measurement, 500 cycles were run at 5 A g^−1^ in order to improve electrolyte access to the micropores. The capacitance was observed to increase up to 5%. Cyclic‐voltammograms (CV) were recorded at various scan rates, galvanostatic charge‐discharge (GCD) at different current densities and electrochemical impedance spectroscopy (EIS) between 500 kHz and 10 mHz with a perturbation amplitude of 5 mV. The specific capacitances (F g^−1^) of a single electrode were calculated from the cyclic‐voltammograms (Equation (7)) and the galvanostatic charge‐discharge (Equation (8)), as follows:

(7) $$C_{CV}=\frac{4I_{\mathrm{cell}}}{\nu \cdot m_{we}}\;\;\left[F\;g^{-1}\right]$$

(8) $$C_{\mathrm{GCD}}=\frac{4\;Q_{\mathrm{tot}}}{\left(\Delta V-IR_{\mathrm{drop}}\right)\cdot m_{we}}\;\left[F\;g^{-1}\right]$$

The relaxation time was calculated from Equation (9):

(9) $$\tau_{0}=\frac{1}{\omega_{0}}\;\;\left[s\right]$$

*ω*_0_ and *C*′′(*ω*) are defined as the following:

(10) $$\frac{dC^{\prime \prime }\left(\omega_{0}\right)}{d\omega }=0$$

(11) $$C^{\prime \prime }\left(\omega \right)=\frac{-Z\left(\omega \right)}{\omega \cdot {\left|Z\left(\omega \right)\right|}^{2}}\;\left[F\right]$$

The following notations are used: *I*_cell_ (mA) cell current, *ν* (mV s^−1^) scan rate of the cyclic‐voltammograms, *Q*_tot_ (*C*) the total charge accumulated in the porous material calculated during the discharge cycle of the cell, Δ*V* (*V*) is the cell voltage, *IR*_drop_ (*V*) is the voltage drop observed when the current is reversed during GCD and *m_we_* (*g*) is the mass of the working electrode only.

The device energy and power densities were calculated from the GCD using Equations (12) and (13):

(12) $$E_{\mathrm{device}}=\frac{E_{\mathrm{discharge}}}{m_{\mathrm{tot}}}=\frac{i\cdot \int_{t_{i}}^{t_{\mathrm{discharge}}}V\left(t\right)\mathrm{dt}}{m_{\mathrm{tot}}}\;\;\;\left[\mathrm{Wh}\;{\mathrm{kg}}^{-1}\right]$$

(13) $$P_{\mathrm{device}}=\frac{E_{\mathrm{device}}}{\Delta t_{\mathrm{discharge}}}\;\left[\mathrm{kW}\;kg^{-1}\right]$$

(14) $$E_{\mathrm{vol}\;\mathrm{device}}=E_{\mathrm{device}}\cdot \rho_{t}\;\;\left[\mathrm{Wh}\;L^{-1}\right]$$

(15) $$P_{\mathrm{vol}\;\mathrm{device}}=P_{\mathrm{device}}\cdot \rho_{t}\;\;\left[\mathrm{kW}\;L^{-1}\right]$$

The following notations are used: *E*_device_ and *P*_device_ are the gravimetric energy and power densities of the full symmetric cell, *E*_vol device_ and *P*_vol device_ are the corresponding volumetric values based on the total volume of the electrode in the cell, *E*_discharge_ is the energy delivered during the discharge of the symmetric device, *i* (mA) is the total cell current, Δ*t*_discharge_ (s) the discharge time and *m*_tot_ (kg) is the mass of the active material in the whole device (working and counter electrode), *V*(*t*) is the voltage function of time, *t_i_* and *t*_discharge_ define the times respectively at the beginning of the discharge (*V* = *V*_cell max_) and at the end (*V* = 0 V).

The pseudocapacitive contributions for both samples were calculated by analyzing first the influence of diffusion on the adsorption kinetics. Assuming a current function of the scan rate in the following form (Equation (16)), a linear regression of the logarithms provides the *b* value corresponding to the slope of Equation (17).

(16) $$i=a\cdot \nu^{b}$$

(17) $$\log\left(i\right)=\log\left(a\right)+b\cdot \log\left(\nu \right)$$

The *b* values were calculated for each sample at potentials step of 0.1 V over the whole voltage window (Figure S9a, Supporting Information). A b value close to 0.5 indicates a solely diffusion limited process whereas solely capacitive processes display a *b* value close to 1. As Figure S9a, Supporting Information, shows *b* values of 0.96 between 0.1 and 1.0 V, the adsorption process is highly dominated by capacitive charge storage. Above 1 V, the *b* value decreases due to a more diffusion‐controlled carbon corrosion limited by the supply of OH^−^ and protons formed in situ.^[^ ^38^, ^72^ ^]^

The pseudocapacitance can then be determined by estimating the relative contributions of diffusion‐ and surface‐controlled capacitive currents to the total capacitance as a function of the scan rate, as reported by Trasatti et al.^[^ ^57^ ^]^ The scan rate dependence of the accumulated charge q on the carbon surface is related to the existence of less accessible surface area such as pores, cracks and areas of lesser affinity with the electrolyte due to surface groups or nanoconfinement.^[^ ^57^ ^]^ As the scan rate increases, the diffusion controlled current becomes limited by the supply of reactants towards the surface and this without affecting the surface‐controlled current observed. Thus, the theoretical maximal capacitance can be extrapolated at a scan rate *ν* = 0 as the electrolyte ions can fully access the micropores and the reactants access the pseudocapacitive active sites. In contrast, as *ν*→∞, only the surface controlled capacitive contribution can be seen.^[^ ^73^ ^]^ The charge q disaccumulated during the gavonostatic discharge was then plotted versus the scan rate *ν* ^−0.5^ (Figures S9b,c, Supporting Information) to double layer charge q_DL_ while the total charge q_T_ was extracted by plotting q^−1^ versus *ν* ^0.5^. The charge from pseudocapacitive processes q_PS_ is then obtained from Equation (18).

(18) $$q_{PS}=q_{T}-q_{DL}$$

## Conflict of Interest

The authors declare no conflict of interest.

## Supporting information

Supporting Information

## Data Availability

Research data are not shared.
