# Supplementary material for: High‐Density Lignin‐Derived Carbon Nanofiber Supercapacitors with Enhanced Volumetric Energy Density
Source: Adv Sci (Weinh). 2021 May 20;8(17):2100016. doi: 10.1002/advs.202100016 (PMC8425891; doi:10.1002/advs.202100016)
Supplement: Supplementary file 1 — Supporting Information [file ADVS-8-2100016-s001.pdf]

© 2021 Wiley-VCH GmbH

ADVANCED SCIENCE
Open Access

Supporting Information

for *Adv. Sci.,* DOI: 10.1002/advs.202100016

High-density Lignin-derived Carbon Nanofiber Supercapacitors with Enhanced Volumetric Energy Density

Servann Hérou, Josh J Bailey, Matt Kok, Philipp Schlee, Rhodri Jervis, Dan J. L. Brett, Paul R. Shearing, Maria Crespo Ribadeneyra, and Magdalena Titirici**

Science Advances
AAAS

advances.sciencemag.org/cgi/content/full/sciadv.[ms.no.]/DC1

Supplementary Materials for

High-density lignin-derived carbon nanofiber supercapacitors with enhanced volumetric energy density

Servann Hérou[1], Josh J Bailey[2,3], Matt Kok[2,3], Philipp Schlee[1], Rhodri Jervis[2,3], Dan J.L. Brett[2,3], Paul R. Shearing[2,3], Maria Crespo Ribadeneyra[1]* and Magdalena Titirici[1,3]*

*Corresponding authors. Email: m.crespo-ribadeneyra@imperial.ac.uk and m.titirici@imperial.ac.uk

This PDF file includes:

Supplementary Text

Figs. S1 to S10

Tables S1 to S2

References (1 to 3) (only referring to papers in the SM)

Other Supplementary Materials for this manuscript include the following:

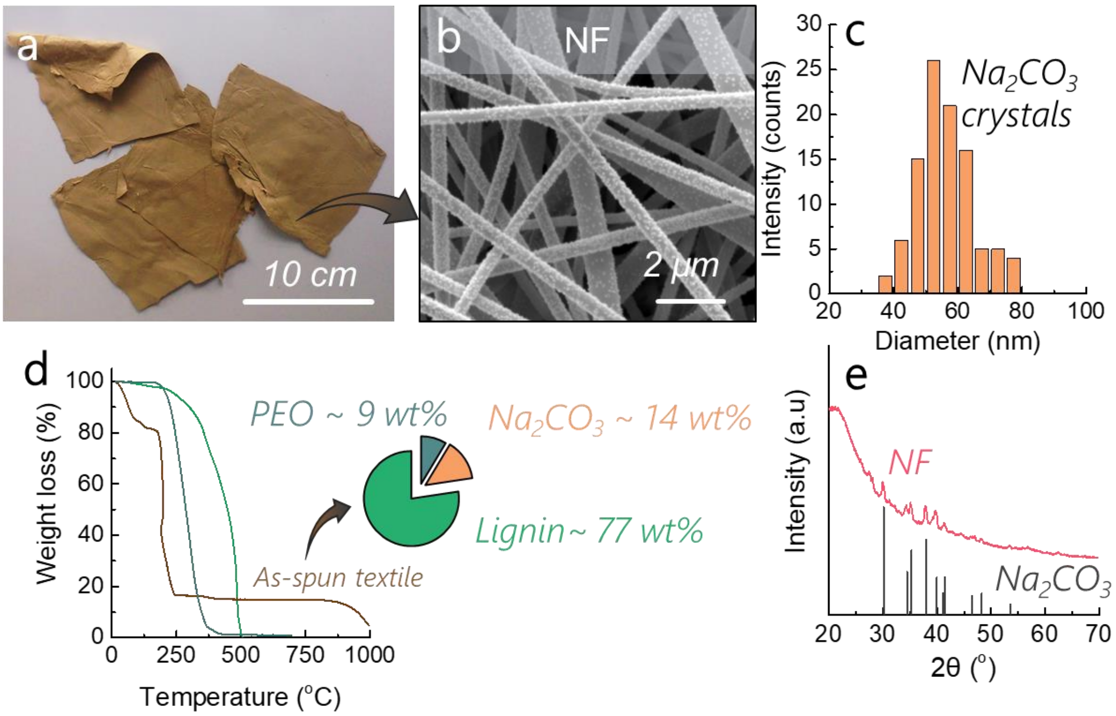

Fig S1. (a) Photograph of the mat after electrospinning; (b) Scanning Electron Micrograph (SEM) of the as-spun mat (NF); (c) Diameter distribution of the sodium carbonate crystals visible on the surface of the nanofibers obtained from micrograph (b); (d) Thermo-gravimetric analysis (TGA) of the as-spun textile, PEO and organosolv lignin in air at 10 °C.min-1; (e) X-Ray Diffraction (XRD) pattern of the as-spun sample NF showing the characteristic peaks of the monoclinic phase of sodium carbonate Na2CO3 (Source: Crystallographic database, Reference code: 00-037-0451).

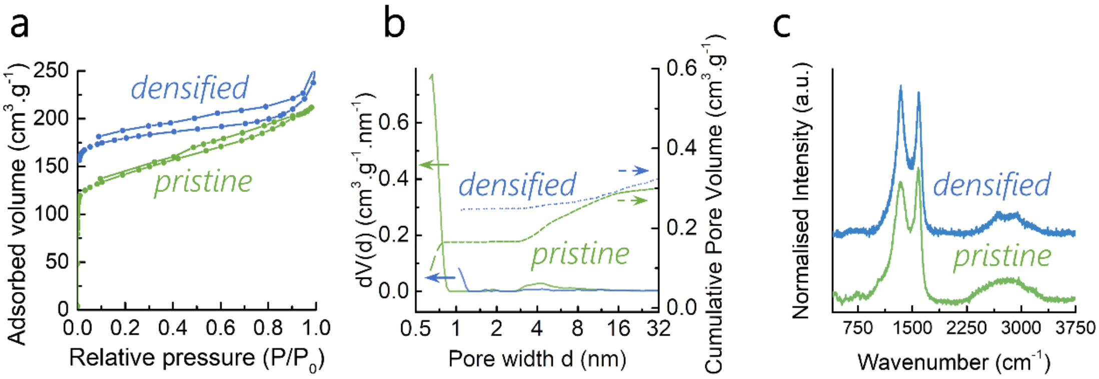

Fig S2. Gas adsorption measurements. (a) N_2 ad-/desorption isotherms at 77 K and (b) Pore size distribution calculated from QSDFT model on the adsorption line and (c) Raman spectra measured with an excitation wavelength of 532nm.

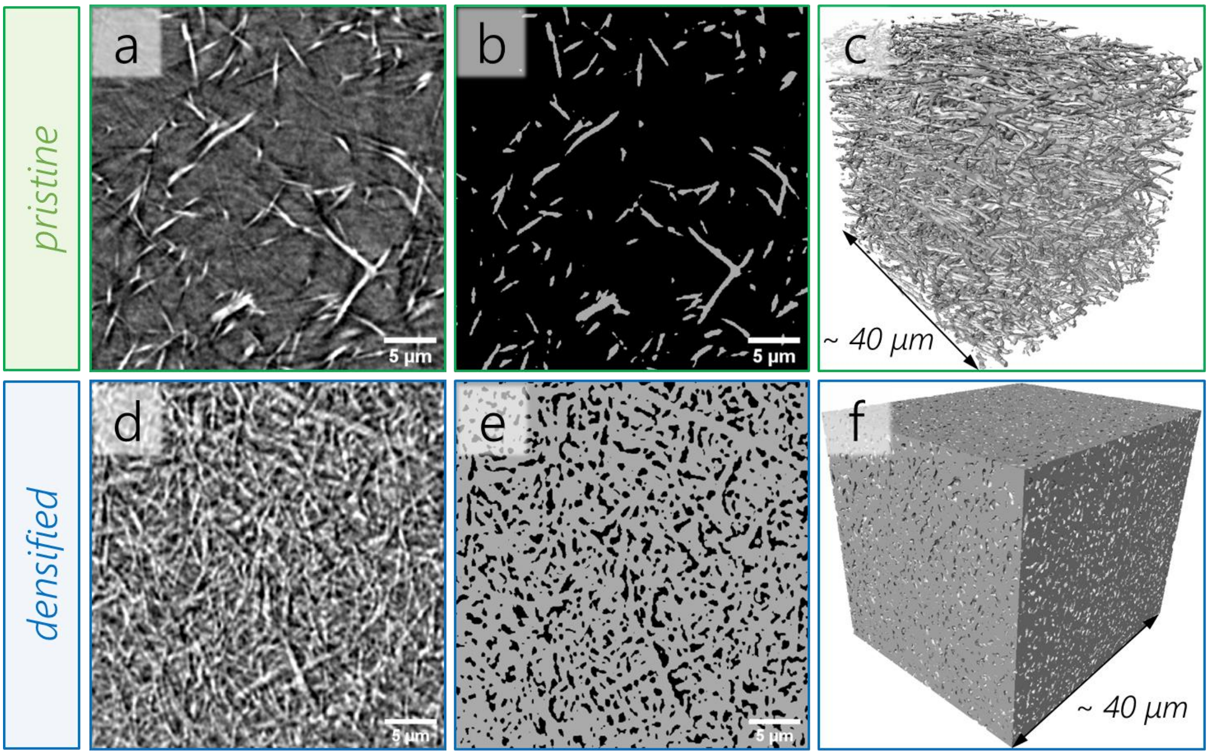

Fig S3. X-ray nano-CT of the pristine (a,b,c) and densified samples (d,e,f) showing a,d) orthoslices from raw tomograms; b,e) Binary segmentations thereof; and (c,f) Surface renderings of the segmented fibers.

Table S1. Porosity data calculated via N_2 adsorption and X-ray nano-computed tomography (nano-CT)

a) Surface properties calculated from the N_2 adsorption line at 77 K and porosity data

	S_{BET} [$m^2.g^{-1}$]	S_{DR} [$m^2.g^{-1}$]	V_{DR} [$cm^3.g^{-1}$]	S_{DFT} [$m^2.g^{-1}$]	$V_{TOT\ DFT}$ [$cm^3.g^{-1}$]	$\frac{V_{MICRO}}{V_{TOTAL}}$ (%)	S_{BJH} [$m^2.g^{-1}$]	V_{BJH} [$cm^3.g^{-1}$]
pristine	531	564	0.201	609	0.301	56	71	0.124
densified	716	781	0.278	830	0.328	76	30	0.117

b) Analysis of "pristine" and "densified" volumes and surface areas from binary segmented tomograms captured by X-ray nano-CT

	Phase	Absolute volume [μm^3]	Volume fraction (-)	Absolute surface area [μm^2]	Volume-specific surface area [μm^{-1}]
pristine	Fibers	3628	8%	33876	0.75
	Pores	41826	92%		
densified	Fibers	36339	79%	91881	2.01
	Pores	9450	21%		

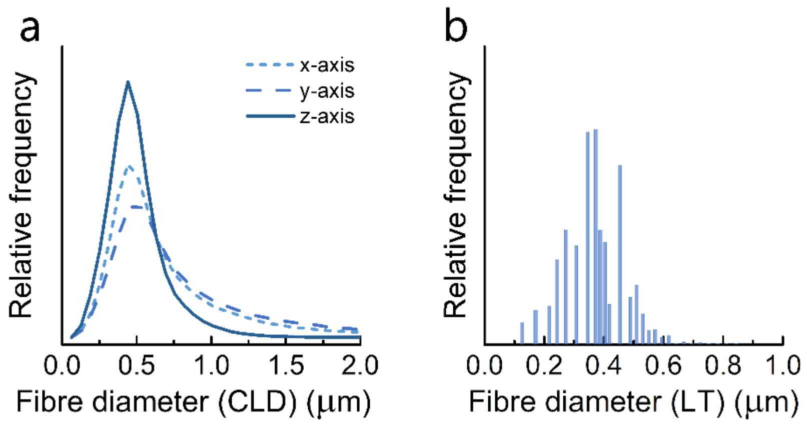

Fig S4. Fiber diameter analysis of densified sample using (a) Three-dimensional Chord Length Distribution (CLD) and (b) Local Thickness methods.

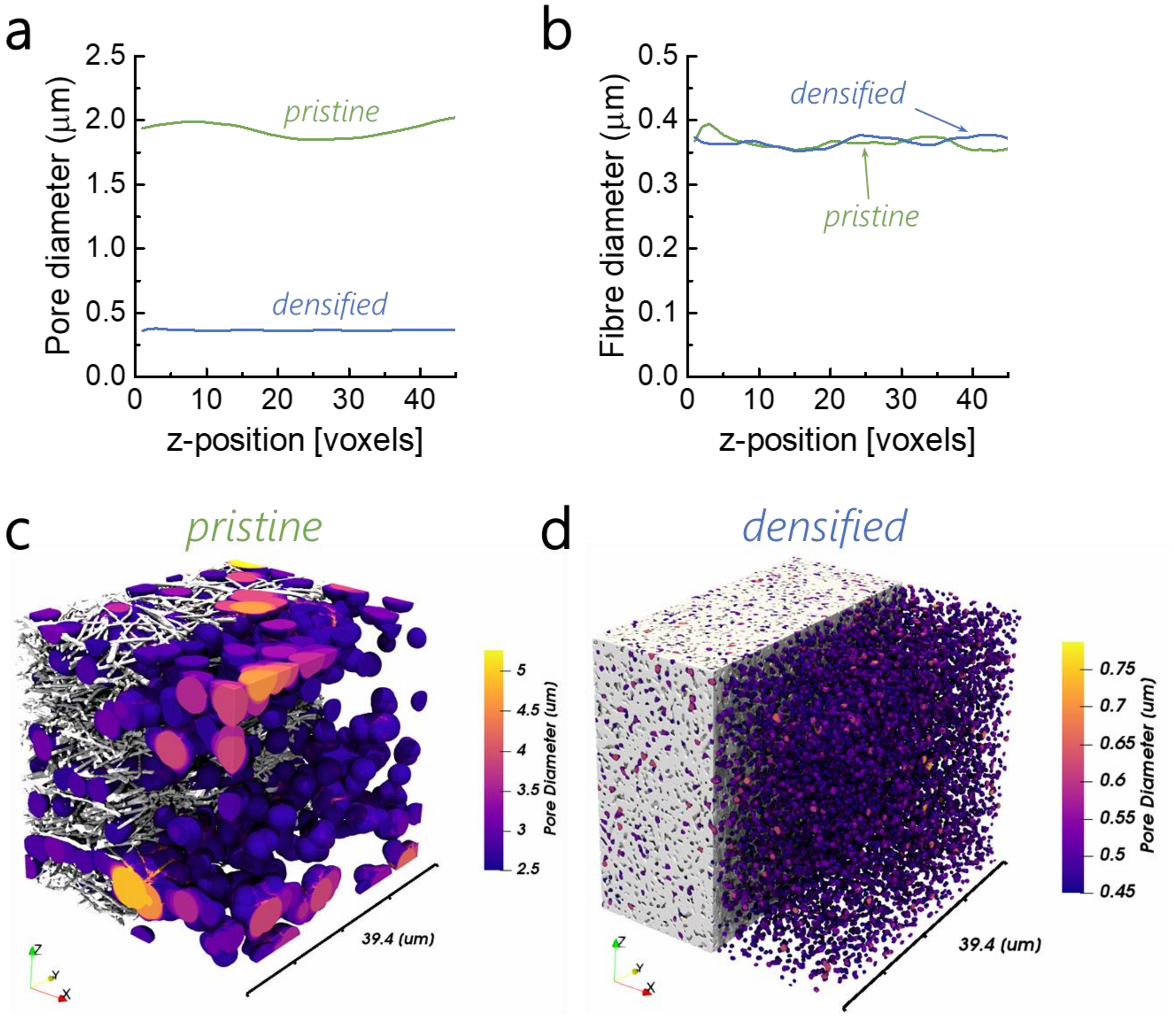

Fig S5. Thickness dependent (a) pore diameter and (b) fiber diameter, determined by local thickness (LT); Local thickness results overlaid on fiber volume renderings for (c) pristine and (d) densified on a large scale (~40 μm).

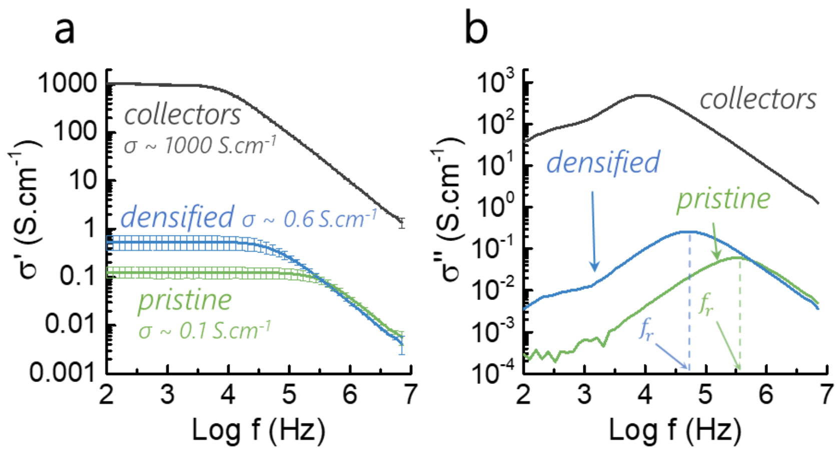

Fig S6. (a) Real component and (b) imaginary component of the electrical conductivity of pristine and densified compared to the current collectors measured by Electrochemical Impedance Spectroscopy (EIS). Calculations detailed in Equations (5) and (6). The relaxation time f_r represents the maximum lag between the oscillating electric field and the charge mobility. Shorter relaxation time are characteristics of more ideally conducting materials, which dissipate less heat when conducting charges.

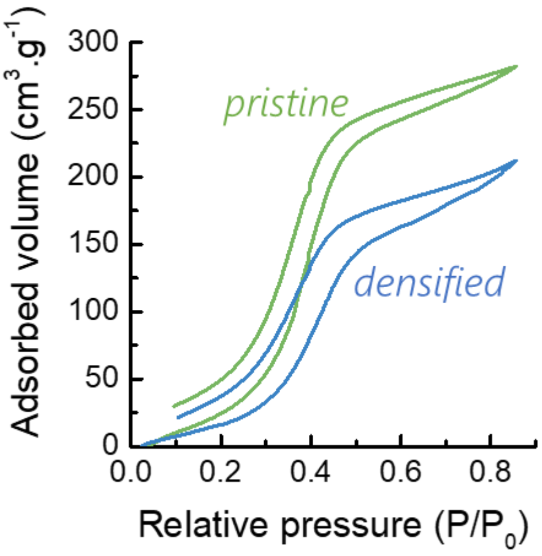

Fig S7. Water adsorption isotherms measured between 10^{-3} and 0.9 P/Po showing the pronounced H_2O adsorption in the micropores at P/P_0 ~ 0.4-0.5.[1]

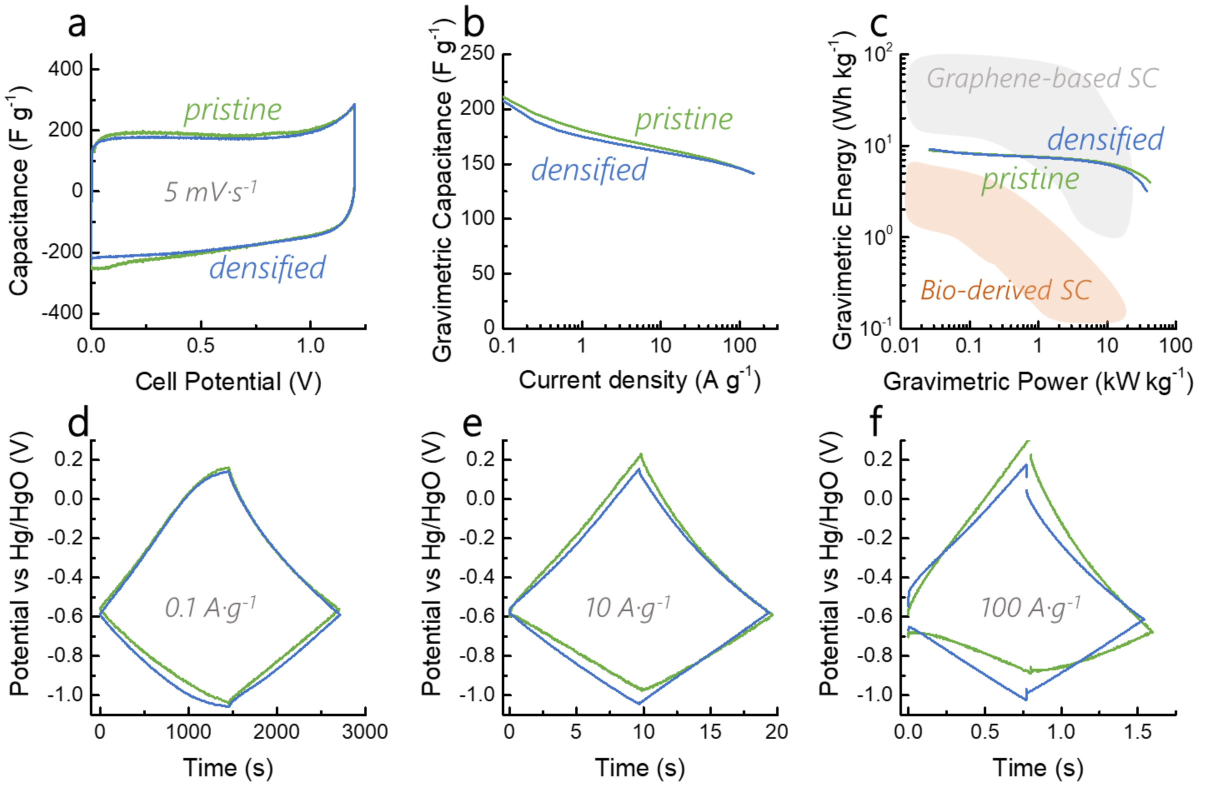

Fig S8. Electrochemical Characterization in aqueous 6M KOH for pristine and densified: (a)-Cyclo-voltammograms measured at 5 mV s^{-1}; (b) Gravimetric rate capability; (c) Gravimetric Ragone plot showing the performances of pristine and densified compared with commercial activated carbon supercapacitors (SC)(see references[2,3]) and the newly developed (<3 years) graphene-based SC (see reference on Figure 4); Galvanostatic charge discharge of the full symmetric supercapacitor at (d) 0.1 A g^{-1}, (e) 10 A g^{-1} and (f) 100 A g^{-1} showing the working and counter electrode polarization becoming more asymmetric at higher rates.

Table S2. Pseudocapacitive contribution to the charge storage

	$\mathbf{C_T}$ [F.g^{-1}]	$\mathbf{C_{DL}}$ [F.g^{-1}]	$\mathbf{C_{PS}}$ [F.g^{-1}]	Pseudocapacitive contribution (%)
pristine	47.6	41.6	6	14.5
densified	46.8	39.6	7.24	18.3

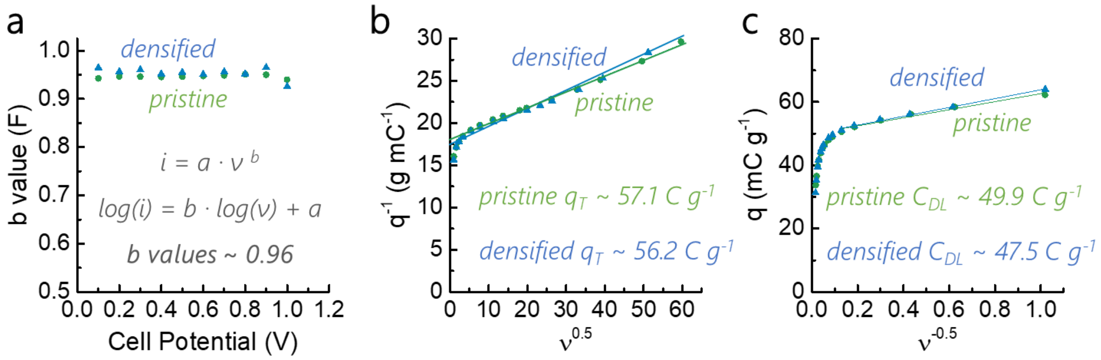

Fig S9. Electrochemical Characterization in aqueous 6M KOH for pristine and densified: (a)-Cyclo-voltammograms measured at 5 mV s^{-1}; (b) Gravimetric rate capability; (c) Gravimetric Ragone plot showing the performances of pristine and densified compared with commercial activated

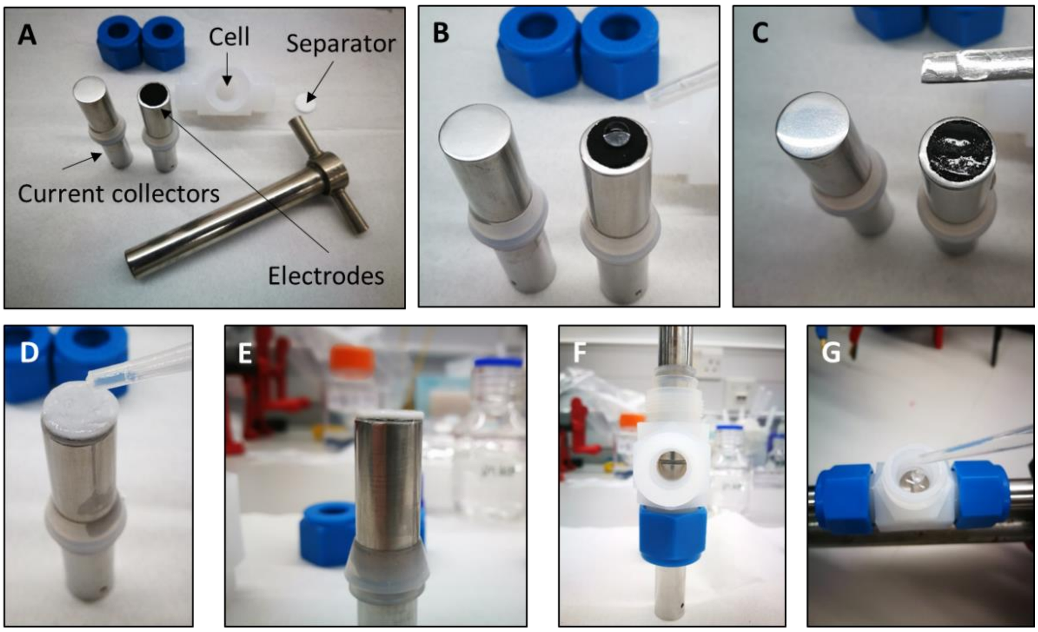

Fig S10. Photographs of the cell assembly showing the assembly steps. (a) Components of the cell; (b) Electrolyte dropping onto the carbon electrode; (c) wetting the electrode with the spatula to ensure maximum penetration of the electrolyte into the micrometer size pores; (d) Soaking of the glass fiber separator with the electrolyte; (e)-(g) mounting the cell by pressing the current collectors in a Swagelok-type cell.

References

[1] L. Liu, S. Johnathan, T. Horikawa, D. D. Do, D. Nicholson, J. Liu, 2017, 250, 64.

[2] Y. Xu, Z. Lin, X. Zhong, X. Huang, N. O. Weiss, Y. Huang, X. Duan, Nat. Commun. 2014, 5.

[3] H. Li, Y. Tao, X. Zheng, J. Luo, F. Kang, H.-M. Cheng, Q.-H. Yang, Energy Environ. Sci. 2016, 9, 3135.
